# Modulation of Speech Motor Learning with Transcranial Direct Current Stimulation of the Inferior Parietal Lobe

**DOI:** 10.3389/fnint.2017.00035

**Published:** 2017-12-15

**Authors:** Mickael L. D. Deroche, Don L. Nguyen, Vincent L. Gracco

**Affiliations:** ^1^Centre for Research on Brain, Language and Music, McGill University, Montreal, QC, Canada; ^2^Haskins Laboratories, New Haven, CT, United States

**Keywords:** speech production, speech perception, sensorimotor adaptation, inferior parietal lobe, transcranial direct current stimulation

## Abstract

The inferior parietal lobe (IPL) is a region of the cortex believed to participate in speech motor learning. In this study, we investigated whether transcranial direct current stimulation (tDCS) of the IPL could influence the extent to which healthy adults (1) adapted to a sensory alteration of their own auditory feedback, and (2) changed their perceptual representation. Seventy subjects completed three tasks: a baseline perceptual task that located the phonetic boundary between the vowels /e/ and /a/; a sensorimotor adaptation task in which subjects produced the word “head” under conditions of altered or unaltered feedback; and a post-adaptation perceptual task identical to the first. Subjects were allocated to four groups which differed in current polarity and feedback manipulation. Subjects who received anodal tDCS to their IPL (i.e., presumably increasing cortical excitability) lowered their first formant frequency (F1) by 10% in opposition to the upward shift in F1 in their auditory feedback. Subjects who received the same stimulation with unaltered feedback did not change their production. Subjects who received cathodal tDCS to their IPL (i.e., presumably decreasing cortical excitability) showed a 5% adaptation to the F1 alteration similar to subjects who received sham tDCS. A subset of subjects returned a few days later to reiterate the same protocol but without tDCS, enabling assessment of any facilitatory effects of the previous tDCS. All subjects exhibited a 5% adaptation effect. In addition, across all subjects and for the two recording sessions, the phonetic boundary was shifted toward the vowel /e/ being repeated, consistently with the selective adaptation effect, but a correlation between perception and production suggested that anodal tDCS had enhanced this perceptual shift. In conclusion, we successfully demonstrated that anodal tDCS could (1) enhance the motor adaptation to a sensory alteration, and (2) potentially affect the perceptual representation of those sounds, but we failed to demonstrate the reverse effect with the cathodal configuration. Overall, tDCS of the left IPL can be used to enhance speech performance but only under conditions in which new or adaptive learning is required.

## Introduction

Speech production depends crucially on hearing one’s own voice. The auditory feedback one receives provides the brain with information of production outcomes which is used to maintain an internal model of the sensorimotor system. One of the ways to study how the auditory, or any sensory system, interacts with the speech motor system is to modify the reafferent (self-generated) feedback and assess the changes induced. In such studies a subject speaks into a microphone which sends the signal to a digital signal processor that manipulates the voice in pseudo real-time (about 10–15 ms delay) and is sent back to the subject via headphones or ear inserts in parallel to one’s own production (Elman, 1981; Kawahara, 1995; Houde and Jordan, 1998, 2002; Jones and Munhall, 2000, 2005; Purcell and Munhall, 2006a,b; Villacorta et al., 2007; Shiller et al., 2009, 2010). If the modified feedback is maintained in a predictable manner over a period of time(10–20 min) subjects alter their speaking in ways that compensate for the perceived error reflecting a short term sensorimotor recalibration. Interestingly, subjects are not often aware of the manipulation, suggesting that the adaptation behavior is not a conscious decision from the subject to oppose the perceived error but rather taps into the tight coupling that exists between speech motor action and sensory feedback. Furthermore, the sensory alteration not only leads to a motor adjustment but also a change in the perceptual representation of the sounds being produced (Villacorta et al., 2007; Shiller et al., 2009, 2010). However, it is still unclear where in the brain this kind of adaptation phenomenon occurs.

The parietal lobe is associated with multisensory integration and sensorimotor transformation. It has been suggested that the parietal lobe is a site for the formation of forward and inverse internal models of sensorimotor control (Wolpert and Kawato, 1998; Desmurget and Grafton, 2000; Della-Maggiore et al., 2004), processes important for speech motor learning. More specifically, the inferior parietal lobe (IPL) is believed to link actions with perception, making it a crucial component in a brain network for imitation and learning (Rizzolatti et al., 2006). Furthermore, the IPL is associated with processing of phonological information of speech (Demonet et al., 1994; Devlin et al., 2003; Deschamps et al., 2014) and contributes to frequency-related transformations of pitch regulation during singing (Zarate and Zatorre, 2008; Zarate et al., 2010). This brain region has also been shown to contribute to speech production under conditions of sensorimotor adaptation. Specifically, lowering the excitability of the IPL in the left hemisphere via repetitive transcranial magnetic stimulation (rTMS) reduced the capacity for sensorimotor learning in response to auditory feedback alteration (Shum et al., 2011). While inhibiting this brain region, which is functionally connected to parts of the inferior frontal and anterior insular cortex, along with the cerebellum, led to a reduction in sensorimotor learning, the question remains whether it is possible to increase sensorimotor learning through increasing excitatory activity. This was one of the motivations for the current study.

However, rather than rely on rTMS, we chose to assess the contribution of the IPL on sensorimotor adaptation using transcranial direct current stimulation (tDCS). Transcranial direct current stimulation uses constant, low current delivered to the brain through small electrodes to modulate cortical excitability (Priori et al., 1998; Nitsche and Paulus, 2000). The direction of effects of tDCS have been shown to be polarity specific with anodal stimulation up-regulating membrane potentials (excitatory action) and cathodal stimulation down-regulating membrane potentials (inhibitory action). In the last decade, tDCS has seen successful application in a number of areas (Rosenkranz et al., 2000; Kwon et al., 2008; Bachmann et al., 2010) including stroke rehabilitation (Schlaug et al., 2008). The stimulation associated with tDCS uses currents on the order of 1–2 milliamps compared to rTMS, which uses currents on the order of 3–5 kiloamps. Therefore, the safety risks are minimal with mild headache and mild burning or itching sensation under the electrodes (Been et al., 2007; Hamilton et al., 2011) reported in rare cases. Increasingly, tDCS is being evaluated as a low-cost, easy to use therapeutic tool to improve clinical depression (Shiozawa et al., 2014), motor skill learning (Buch et al., 2016), and cognitive function (Jacobson et al., 2012). For motor learning and motor adaptation, anodal stimulation has been shown to have some positive effects especially over motor cortex although a number of issues have been identified including reproducibility, inter-subject variability, and the lack of standardized protocols (see Buch et al., 2016 for an extensive overview). Here we took the opportunity to evaluate the effectiveness of tDCS for sensorimotor adaptation for speech production to examine in more detail the findings from a previous study using rTMS over the inferior parietal cortex (Shum et al., 2011). One of the observations from the Shum et al. (2011) study using off-line inhibitory rTMS, was that while inhibitory stimulation reduced the adaptation response, it had no measurable effect on unaltered speech production (when the subjects’ auditory feedback was not manipulated). Interestingly, the stimulation site was a region in the secondary somatosensory cortex that is normally not active or even deactivated (Dhanjal et al., 2008; Geranmayeh et al., 2012, 2014; Seghier and Price, 2012) during most speech production tasks and seemingly becomes active under novel orofacial or speech conditions (oral movements, pseudoword production). If the IPL is part of a learning network as the literature suggests, then it may only be engaged under learning or adaptive conditions. This prediction was tested in the current study.

For the current study we used an adaptation paradigm in which the first major resonance of the vocal tract (the first formant or F1) associated with the vowel /e/ in the word “head” was increased. The increase in F1 frequency under normal speech production is inversely associated with the height of the tongue in the oral cavity. As a result, the feedback is reflecting a lower tongue position which results in an adaptive response consistent with raising the tongue and a decrease in F1 to adjust to the illusion of an incorrect spatial position of the tongue. We estimated the change in speech motor space from the change in the F1 frequency and evaluated the change in discrimination of the same vowel to assess change in perceptual space. There were four experimental groups of subjects. In the Anodal group, subjects received tDCS to enhance excitability of the IPL. In the Cathodal group, subjects received tDCS to diminish excitability of the IPL. In the Sham group, subjects received fake stimulation as a control condition for possible placebo effects. Under conditions of altered auditory feedback, it was hypothesized that (1) subjects in the Anodal group would exhibit the strongest adaptation effect in F1 and potentially largest changes in the perceptual dimension between /e/ and /a/, (2) subjects in the Cathodal group would exhibit the weakest adaptation effect in production and potentially the smallest changes in perceptual representation, (3) subjects in the Sham group would exhibit a typical adaptation effect (with a magnitude in between the first two groups). In the last group of subjects, anodal tDCS was applied but contrary to the three other groups, there was no feedback alteration. Here we examined whether increased excitability to the IPL region in the absence of adaptive learning, would show an effect. Observing no effect would be consistent with our hypothesis that the IPL is only engaged under conditions which required a change in the internal model. Finally, the extent to which changes in sensorimotor learning persist over time largely remains an open question. In this study, we invited subjects for retesting on subsequent days to probe for long-lasting carry-over effects of the experimental manipulations performed during the first round of testing.

## Materials and Methods

### Participants

A power analysis was performed to estimate sample sizes. The primary focus was on the effect of tDCS, and therefore considered the three groups Anodal, Cathodal, and Sham. The effect of tDCS on adaptation to altered feedback was expected to be relatively weak: assuming an effect size of 0.3–0.35, an *a priori* power of 0.8 required a sample size of 45–60 subjects for this between-subject factor measured over 90 trials (i.e., considering the hold phase only) with a correlation among repeated measures of 0.5. Within this range, we settled for 18 subjects in each group, for a total of 54 subjects. A secondary analysis focussed on the adaptation phenomenon, i.e., the comparison between Anodal and Anodal-0 groups. The adaptation effect (relative to the non-altered feedback condition) was expected to be strong. Assuming an effect size of 0.8, an *a priori* power of 0.95 required a sample size of 14 subjects (i.e., only 7 subjects in each of the two groups) with 90 measurements correlated at 0.5. Given that 18 subjects were already assigned to the Anodal group, we settled for 16 subjects for the Anodal-0 group, simply to provide a more balanced size for the last group. Thus, a total of 70 subjects participated in this study. They were allocated to four groups in a random manner except for our attempt to equate the female-to-male ratio among the groups. There were 18 subjects (13 females, 5 males) in the Anodal group; 18 subjects (13 females, 5 males) in the Cathodal group; 18 subjects (12 females, 6 males) in the Sham group. The remaining 16 subjects (10 females, 6 males) were in the Anodal-0 group in which they were subjected to anodal stimulation but no feedback manipulation. All subjects were healthy adults between 18 and 38 years old, with a mean [and standard deviation] of 23.4 [5.5], 22.0 [2.3], 23.9 [4.6], 23.6 [3.8], respectively, in the Anodal, Cathodal, Sham, and Anodal-0 group. They were mostly university students, native speakers of English, with no history of neurological, speech, or hearing disorders. Four subjects were left-handed (two in the Cathodal group and two in the Sham group) and were included because analysis of their data did not reveal any different pattern from the rest of their group. Prior to testing, all participants provided informed consent in accordance with the ethics principles approved by the Institutional Review Board of the Faculty of Medicine at McGill. They were paid for their participation.

### Stimuli

The stimuli used in the perceptual task were generated from a synthetic continuum with 10 steps in which F1 and F2 were varied from the vowel /e/ to /a/ embedded in the word “head.” These steps corresponded to mean F1 values of 553.5, 581.4, 601.7, 618.3, 629.4, 640.0, 647.7, 657.2, 664.0, and 676.6 Hz, spanning a 123-Hz range. These steps also corresponded to mean F2 values of 1681.9, 1668.6, 1654.8, 1643.1, 1632.0, 1620.6, 1607.8, 1594.2, 1574.2, and 1554.8 Hz, spanning a 127-Hz range. Their duration was 200 ms and they were all equalized at 70 dB SPL.

In the production task, the stimuli heard by subjects were their own voice passed through the digital signal processor for formant manipulation. The output signal was then mixed with speech-shaped noise presented at 70 dB SPL. In the light of previous studies (e.g., Shiller et al., 2009), this level was deemed sufficient to mask the subject’s perception of their own air/bone conducted speech, while allowing for clear perception of the modified feedback signal.

### Protocol

**FIGURE 1:**
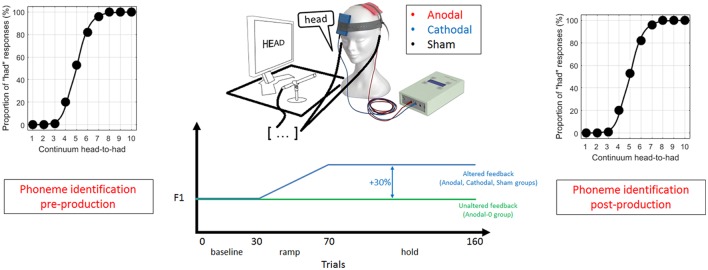
Schematic of the experimental design in three phases: (1) a pre-production phoneme identification task, (2) recording of 160 productions of the word “head” with altered or unaltered feedback (depending on trial number and experimental group) during which transcranial direct current stimulation (tDCS) was on, and (3) a post-production phoneme identification task.

Each session consisted of three parts, which are illustrated in **Figure 1**. First, subjects were presented with 100 stimuli (10 repetitions of the 10 steps spanning the continuum) presented randomly (with a different randomization for each subject), and were asked on each trial to indicate, by clicking with a mouse, whether it sounded more like “head” or “had.” This forced-choice perceptual task enabled the location of the phonetic boundary between /e/ and /a/, intrinsic to an individual subject.

Second, subjects were asked to speak the word “head” 160 times in a microphone while hearing their own voice through ear inserts. Each production was cued by the presentation of the word “head” on a monitor displayed at a distance of 0.5 meters, and subjects were instructed to speak immediately after seeing the word which remained on-screen for 1.2 s, followed by a 1.2 s pause.

For the subjects allocated to the Anodal, Cathodal, and Sham group, the auditory feedback of their own voice could be altered depending on the trial. The first 30 productions constituted the baseline phase, where the auditory feedback was unaltered. This phase obtained a reliable estimate of the mean F1 under normal feedback conditions. The next 40 trials constituted the ramp phase, where the F1 of the auditory feedback was progressively increased up to a maximum shift of 30%. The last 90 trials constituted the hold phase where the shift was maintained at 30%. It was expected that subjects would lower their F1 to oppose the F1 shift in auditory feedback. In contrast to the three other groups, subjects allocated to the Anodal-0 group listened to their own voice with unaltered feedback (through the same apparatus) throughout the 160 trials. This condition was used to control for any effect intrinsic to the anodal tDCS and to evaluate the excitatory effects of IPL stimulation in the absence of feedback manipulation.

Subjects were not aware of the experimental group they were allocated to. They were aware that their voice was recorded and passed through a machine that could manipulate it, but they were not told about the nature of the manipulation that was to occur (i.e., whether it was about pitch, loudness, timbre or any other percept). They were simply instructed to speak clearly and sufficiently loud by tracking their voice intensity visually by a level meter (a rising vertical bar) displayed on the monitor in front of them. There was no systematic assessment of whether the subject was eventually aware of the manipulation, but informal reports ranged from “I did not hear anything strange” to “my voice sounded a little weird.”

The third part of the protocol was a direct replication of the first part, i.e., the perceptual task, meant to evaluate whether the phonetic boundary between “head” and “had” had changed relative to the pre-production boundary, and whether this change depended on the experimental group. The total duration of an experimental session was 40–50 min. It included description of the protocol, completion of the consent form and a short questionnaire about the musical background, hand preferences, and medical history, which took about 10 min.

To investigate the effect of the tDCS on sensorimotor adaptation within the same subjects, and potentially probe for effects carrying over to the following days, subjects in the Anodal, Cathodal, and Sham groups were invited to come back for a second round of testing. Subjects in the Anodal-0 group were not invited on the basis that this condition ought not to have produced any sensorimotor adaptation (since the auditory feedback was unaltered). Out of the 18 subjects initially allocated to each group, 10 subjects in the Anodal group, 8 in the Cathodal group, and 9 in the Sham group returned for the second session. The protocol in round 2 was identical to that in round 1 with the exception that tDCS was not used. In other words, all three groups went through the same experimental condition in which the F1 manipulation in auditory feedback was expected to lead to sensorimotor adaptation.

### Stimulation

Before beginning the recordings, an experimenter set up the tDCS equipment on the head of the subject. None of the subjects had ever been exposed to the tDCS technique before, nor were they aware that different types of stimulation existed. The experimenter, however, was aware of both the feedback manipulation and the tDCS settings, so this was not a double-blinded study. The first electrode (to which polarity refers, area = 25 cm^2^) was placed over P3 of the international 10–20 system for EEG electrode placement. This location overlies the posterior parietal cortex in close proximity to the intraparietal sulcus. The reference electrode (area = 25 cm^2^) was placed over the right supraorbital region. The experimenter started the current stimulation just before launching the adaptation task and ended it manually after the adaptation task was completed (6.4 min later). There was a 1-s rise and fall time gating the whole duration and the current intensity was set at 1 milliamp.

### Equipment

The study took place at the Center for Research on Brain, Language, and Music affiliated with McGill University in Montreal. All subjects were tested while seated in a sound attenuating testing room (Industrial Acoustics Company, Bronx, NY, United States). The microphone used for the recordings was a ME-66 Sennheiser condenser microphone which sent the input signal to a preamplifier (model 302, Symetrix, Mountlake Terrace, WA, United States) that amplified it to line level. The signal was then split into two channels. The first channel carried the unprocessed signal, which was low-pass filtered at 22 kHz and digitized at 44.1 kHz and 16-bit resolution by an analog-to-digital converter (Transit, M-Audio, Irwindale, CA, United States). This converter was attached via USB to a Toshiba laptop computer and the digitized audio signal was captured into Matlab. The second channel was sent to a digital signal processor (VoiceOne, manufactured by T.C. Electronic, Denmark) that served to manipulate the formant frequencies of the input signal. The delay introduced by signal processing was about 10–15 ms. The output signal was then sent back to the subject through ear inserts.

## Data Analysis

### Production Data

The portion of interest in each recording of “head” was the vowel /e/. To extract formant values that were as accurate as possible, it was best to disregard the “h” portion and the “d” portion of the recordings. This was achieved by (1) extracting the broadband temporal envelope of the signal by using the absolute value of its Hilbert transform, (2) smooth it in time with a 5-ms pseudo-Gaussian window, (3) picking the maximum of the smoothed Hilbert envelope and finding the two instants corresponding to half of this value. This automatic procedure failed in very rare cases where something peculiar occurred during the recording (e.g., a sneeze, a throat clearing, an external noise) or when subjects did not speak at the right times (either too early or too late). For this reason, every single recording was screened visually and those recordings were either delimited manually or (as in the case of mistimed productions) simply discarded from any subsequent analysis.

A Matlab routine was then used to analyze the recordings. This Matlab routine called Praat, a speech analysis and synthesis software (Boersma and Weenink, 2013) which estimated F1, by choosing a robust method with a 40-ms window, a pre-emphasis of 50 Hz, and tracking it around 550 Hz. These formant values were stored dynamically (i.e., keeping the full pattern over time) but the results of the present analysis focussed on the average of the 50-ms middle portion of each pattern. In practice, this provided slightly more reliable estimates than means which could be affected by early or late portions related to coarticulation. Note that the average of the 50-ms middle portion led to practically the same results as medians.

### Perception Data

In each experimental session, the proportions of “had” responses were summed over the 10 repetitions of each step in the continuum to form a typical psychometric function representing the phonetic boundary between /e/ and /a/. Those data were then fitted using the maximum-likelihood technique described by Wichmann and Hill (2001a,b). The underlying shape was modeled as a logistic function. A priori distributions for the logistic parameters served to guide the fitting procedure. Even though the lower and upper bounds were very close to 0 and 100%, they were given a Gaussian prior with a mean of 0.5% and a standard deviation of 2% to allow for occasional inattention mistakes. The parameter related to the inflection point had a Gaussian prior with a mean of 5.5 (the middle of the continuum) and a standard deviation of 2. The parameter related to the slope had a Gaussian prior with a mean of 0.7 and a standard deviation of 0.5. Note that these distributions were chosen to be sufficiently broad to encompass all the different subjects. Once logistic fits were obtained, thresholds and slopes were extracted at the 50% point and formed the datasets on which statistical analyses were based. Note that other analyses were reiterated with inflection point and sigma as dependent variables (rather than thresholds and slopes) but they led to similar results qualitatively because the lower and upper asymptotes were very close to floor and ceiling.

### Statistical Analysis

For the production data, the primary hypothesis was tested using a mixed analysis of variance (ANOVA) on the normalized F1 values, with one between-subject factor “Group” (with four levels: Anodal, Cathodal, Sham, and Anodal-0) and one within-subject factor “Phase” (with three levels corresponding to the average of baseline, i.e., trials 01–30, ramp, i.e., trials 31–70, and hold phase, i.e., trials 71–160). The expected interaction between phase and group was further examined by testing the simple effect of phase for each group, and the simple effect of group for the ramp and hold phase. Differences between groups over the baseline phase were treated initially from the F1 values expressed in Hz. Finally, pairwise comparisons were performed, and reported with and without Bonferroni corrections for multiple comparisons, to further describe the cause of the simple effects. In round 2, a similar ANOVA was performed, but Group had only three levels (Anodal, Cathodal, and Sham). To test specifically for the effect of tDCS as a within-subject factor, a different ANOVA was performed with the subset of subjects who took part in both rounds. The expected interaction between round and group was further examined by testing the simple effect of round in each group.

For the perception data, the primary hypothesis was tested using a mixed ANOVA with one between-subject factor “Group” (with four levels: Anodal, Cathodal, Sham, and Anodal-0) and one within-subject factor “Session” (with two levels: pre-versus post-production). This was done separately for two parameters of the psychometric function depicting the /e/-/a/ phonetic boundary: the 50% threshold and the slope at threshold. To test specifically for the effect of tDCS as a within-subject factor, a different ANOVA was performed. Group had only three levels (Anodal, Cathodal, and Sham) and two within-subject factors were considered “Session” and “Round.” Again, this was done separately for thresholds and slopes.

## Results

### Production in Round 1

**FIGURE 2:**
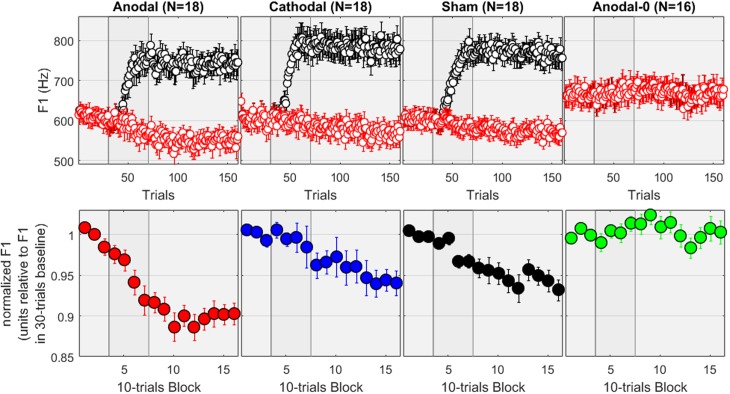
**(Top)** F1 values in Hz measured across 160 recordings in each group. The red dots correspond to the input signals while the black dots are the values extracted from the auditory feedback, which for the first three columns was progressively altered during the ramp phase and held at +30% during the hold phase. Subjects in the Anodal-0 group (most right panel) had unaltered feedback. **(Bottom)** F1 values normalized by their baseline average for each subject, pooled in 10-trial bins.

The top panels of **Figure 2** show the F1 values, expressed in Hz, for the four groups of subjects across the 160 trials. The red dots correspond to the input signals (i.e., the recordings) while the black dots are the values extracted from the auditory feedback. It can be verified that, for the first 30 trials (baseline), the feedback of F1 was intact, whereas it progressively increased during the 40 trials of the ramp phase, and was held for the 90 trials of the hold phase at +30% of its value. Expressed in Hz, this relates to 165–180 Hz above the F1 values produced over 550–600 Hz. For the subjects allocated to the Anodal-0 group, the feedback was always intact, as illustrated by the overlap between black and red dots (most right panel). A repeated-measures ANOVA was conducted on the F1 values averaged over the baseline phase (in Hz, as represented on the top panels). There was no effect of group [*F*(3,69) = 1.8, *p* = 0.151], suggesting no initial difference induced by speaking characteristics of individual subjects.

The bottom panels of **Figure 2** show the F1 values, normalized by the average of the values measured from trial 01 to 30, and pooled within 10-trials bins. First, the most striking observation is that nothing happens for the Anodal-0 group: the normalized F1 fluctuates between ±2%. In contrast, for the three other groups, the normalized F1 decreases in opposition to the F1-shift. This is the adaptation effect, which amounted here to about 5% for the subjects allocated to the Cathodal and Sham groups, and about 10% for the subjects allocated to the Anodal group.

To evaluate these patterns more rigorously, the mixed ANOVA revealed a main effect of group [*F*(3,66) = 8.6, *p* < 0.001], a main effect of phase [*F*(2,132) = 37.7, *p* < 0.001], and an interaction [*F*(6,132) = 7.2, *p* < 0.001]. The effect of group was significant in both the ramp [*F*(3,66) = 4.5, *p* = 0.006] and the hold phase [*F*(3,66) = 10.4, *p* < 0.001]. More specifically, pairwise comparisons between the groups revealed that during the ramp phase, Anodal-0 differed from Anodal (*p* = 0.001) but not from Cathodal (*p* = 0.633) or Sham (*p* = 0.159). Cathodal and Sham did not differ from each other (*p* = 0.333) but both tended to differ from Anodal (*p* = 0.004, and *p* = 0.052). Note that the comparison Anodal vs. Cathodal would survive Bonferroni corrections for six multiple comparisons, but the comparison Anodal vs. Sham would largely fail to reach significance. During the hold phase, Anodal-0 differed from the three other groups (*p* < 0.001, *p* = 0.009, and *p* = 0.003; even though the Anodal-0 vs. Cathodal comparison would not survive Bonferroni corrections); and Anodal differed from Cathodal (*p* = 0.004) and Sham (*p* = 0.013, a difference which would not survive Bonferroni correction) which did not differ from each other (*p* = 0.683). In other words, adaptation occurred over the hold phase for all three experimental groups compared to the Anodal-0 group, and the adaptation effect was stronger in the Anodal group than in the Cathodal or Sham groups.

Additionally, we examined the effect of phase in each group. For the Anodal-0 group, all three phases were similar [*F*(2,65) < 0.1, *p* = 0.935], reflecting that there was no change in F1 as long as its feedback was not manipulated. For the three other groups, phase had a significant effect [*F*(2,65) = 29.1, 9.3, and 8.9, *p* < 0.001 in each case, respectively, for Anodal, Cathodal, and Sham]. Pairwise comparisons revealed that, for the Anodal group, F1 was lower over the ramp than over the baseline (*p* < 0.001), and lower over the hold than the ramp phase (*p* < 0.001). For both Cathodal and Sham groups, F1 did not differ between baseline and ramp (*p* > 0.197) but was lower over the hold phase than over either of baseline or ramp (*p* < 0.003). Note that all these comparisons would survive Bonferroni corrections. In other words, adaptation occurred for the three groups in which F1 was manipulated, but it occurred sooner (i.e., already observable over the ramp phase) for the Anodal group than for either the Cathodal or the Sham group.

### Production in Round 2

**FIGURE 3:**
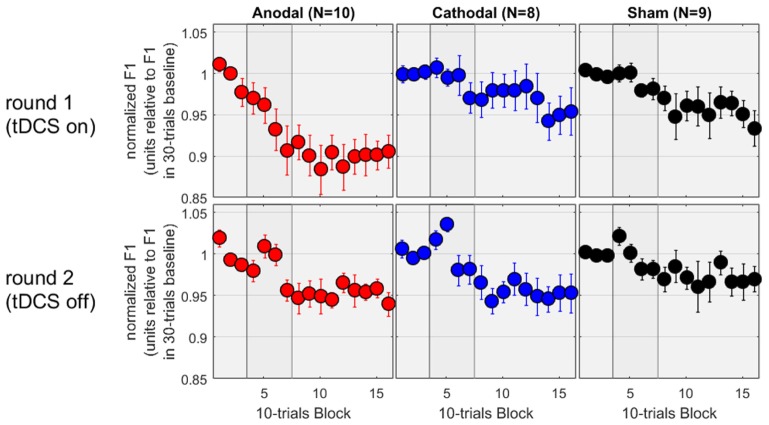
Normalized F1 values for a subset of subjects who came back a second time for testing without tDCS.

In the second round of testing, the tDCS was absent. Except for this difference, the equipment and environment were identical. Thus, the allocation to “Anodal,” “Cathodal,” and “Sham” all referred to the same experimental condition and subjects from the first round. We therefore expected to observe a similar adaptation effect in all three groups, and this is exactly what we found. On the bottom panels of **Figure 3**, the patterns are noisier (which is to be expected given the smaller sample sizes) but all three groups showed a decrease in F1 between 3 and 5%. To evaluate these patterns rigorously, the mixed ANOVA revealed no main effect of group [*F*(2,24) = 0.4, *p* = 0.696], a main effect of phase [*F*(2,48) = 13.9, *p* < 0.001], and no interaction [*F*(4,48) = 0.3, *p* = 0.873]. As illustrated on the bottom panels, the effect of phase reflected that F1 values over the hold phase were lower than those over the baseline or over the ramp (*p* = 0.002 and *p* < 0.001, respectively, both surviving Bonferroni corrections), while F1 values did not differ between baseline and ramp. Thus, adaptation occurred over the hold phase, similarly in all groups.

Since these subjects had taken part in both rounds, we could also evaluate the effect of tDCS as a within-subject factor. The results for the first round on this subset of subjects are shown on the top panels of **Figure 3**, and exhibit a pattern very similar to the one analyzed and discussed in the previous section, namely an adaptation effect of about 10% in the Anodal group, but only 3–5% in the Cathodal and Sham group, measured over the hold phase. We focussed on the first five blocks of the hold phase (roughly to examine the first half where the differences between Anodal and the other two groups seemed qualitatively larger than in the second half, during the first round) and performed a mixed ANOVA with group as between-subjects factor and round as within-subjects factor. There was no main effect of group [*F*(2,24) = 2.3, *p* = 0.126], no main effect of round [*F*(1,24) = 2.1, *p* = 0.156], but a significant interaction [*F*(2,24) = 3.6, *p* = 0.042]. Delving further into this interaction, F1 values were lower in round 1 than round 2 for the Anodal group [*F*(1,24) = 8.9, *p* = 0.006], whereas round had no effect for the Cathodal [*F*(1,24) = 0.8, *p* = 0.380] or Sham [*F*(1,24) = 0.4, *p* = 0.515] groups. The choice of the first five blocks of the hold phase may seem arbitrary. So, we reiterated this analysis by including more and more blocks. The interaction between round and group remained significant, by considering any block between 05 and 13, i.e., including the last three blocks of the ramp phase and the first six blocks of the hold phase (90 trials in total with a maximum *p*-value of 0.031), but it was short of significance either by including block 04 or by including block 14, 15, and 16. In other words, the evidence that the tDCS augmented the adaptation phenomenon for the 10 subjects of the Anodal group (while it did not have any effect for the 8 and 9 subjects of the Cathodal and Sham groups) was most easily observable in the late part of the ramp phase and the early part of the hold phase.

### Perception in Round 1

**FIGURE 4:**
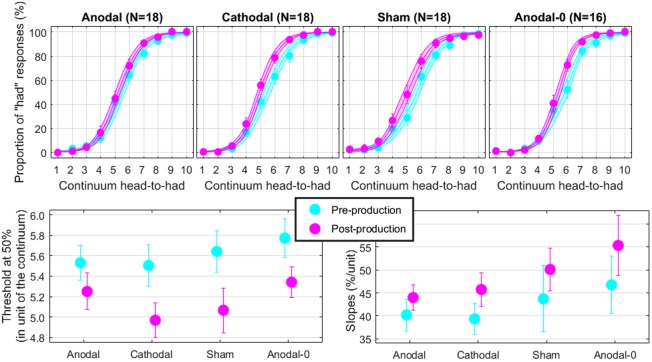
Psychometric functions reflecting the phonetic boundary between “head” and “had” in each experimental group **(top)**. Thresholds **(bottom-left)** and slopes **(bottom-right)** extracted at the 50% point from the logistic fits.

The top panels of **Figure 4** show the proportions of “had” responses along the continuum ranging between “head” and “had.” When the word “head” was presented (stimulus 01 on the abscissa), subjects responded “head” more than 98% of the time. Similarly, when the word “had” was presented (stimulus 10 on the abscissa), subjects responded “had” more than 98% of the time. In between these two categories, the proportion of “had” responses increased following a typical psychometric shape, which was very well fitted with logistic functions shown as lines (means) and areas (one standard error of the mean) in each group. The thresholds extracted from these fits at the 50% point are represented on the bottom-left panel and the slopes at the 50% point represented on the bottom-right panel. Qualitatively, two observations are striking: thresholds were shifted leftward and slopes were steeper in the post-production session compared with the pre-production session. The mixed ANOVA (whose results are displayed in **Table 1**) confirmed these two observations, i.e., a main effect of session for both threshold and slope, but no interaction with group.

**Table 1:** Statistical results of a mixed analysis of variance (ANOVA) on thresholds and slopes for the 70 subjects that participated in round 1.

Factors	Round 1 (*N* = 70)	
	
	Thresholds	Slopes
Group	*F*(3,66) = 0.6	*F*(3,66) = 1.1
	*p* = 0.631	*p* = 0.337
Session	*F*(1,66) = 35.5	*F*(1,66) = 4.4
	*p* < 0.001^∗^	*p* = 0.040^∗^
Group × Session	*F*(3,66) = 0.8	*F*(3,66) = 0.1
	*p* = 0.514	*p* = 0.960


*Group refers to the between-subject factor with four levels (Anodal, Cathodal, Sham, and Anodal-0). Session refers to the within-subject factor with two levels (pre- vs. post-production). ^∗^*p* < 0.05.*

Individual subjects have different phonetic boundaries. Some are closer to “head” and some are closer to “had,” and subjects were randomly assigned to each group before knowing their phonetic boundary. As a consequence, there was no reason that forced all four groups to exhibit the same pre-production thresholds. Simply, we assumed that those pre-production differences between groups would disappear with enough subjects. This assumption was met since pre-production thresholds differed by less than 0.3 among the four groups [*F*(3,69) = 0.4, *p* = 0.762] and were in fact very close to 5.5, i.e., right in the middle of the continuum. In the post-production session, subjects shifted their boundary closer to the “head” percept, by 0.46 on average across all subjects. Critically, since this shift occurred for the Anodal-0 group, it cannot be due to the F1 manipulation in auditory feedback and therefore must be due to the mere repetition of the word “head” shrinking its own perceptual category. Notably, this analysis did not reveal an interaction between group and session, providing no support for our hypothesis that Anodal tDCS would have induced the largest changes in perceptual representation of those sounds.

### Perception in Round 2

The subset of subjects who came twice enabled us to examine the effect of the tDCS on the leftward shift in phonetic boundary as a within-subjects factor. Since there was no tDCS involved in round 2, a leftward shift could be expected of similar magnitude in all three groups. More particularly, we were interested in comparing the leftward shift for the Anodal group between the two rounds. If it were significantly smaller in round 2 than in round 1, then anodal tDCS could be said to have enhanced the pull toward “head.”

**FIGURE 5:**
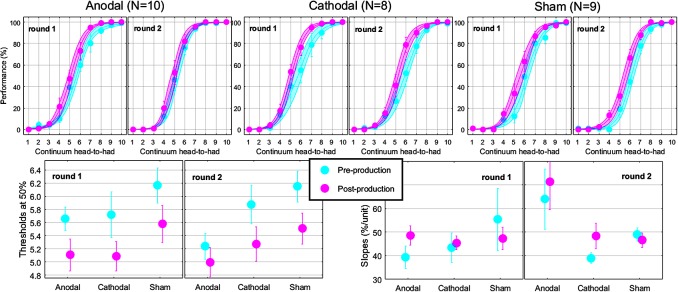
Psychometric functions reflecting the phonetic boundary between “head” and “had” in each experimental group **(top)**. Thresholds **(bottom-left)** and slopes **(bottom-right)** extracted at the 50% point from the logistic fits.

The top panels of **Figure 5** show the psychometric functions in both rounds for the subset of subjects who came twice. As before, these data were very well fitted with logistic shapes enabling accurate extraction of thresholds (bottom-left panels) and slope (bottom-right panels) at the 50% point. The mixed ANOVA was conducted on thresholds and slopes, whose results are displayed in **Table 2**.

**Table 2:** Statistical results of a mixed ANOVA on thresholds and slopes for the 27 subjects that participated in both rounds.

Factors	Round 1 and 2 (*N* = 27)	
	
	Thresholds	Slopes
Group	*F*(2,24) = 2.1	*F*(2,24) = 1.4
	*p* = 0.144	*p* = 0.260
Round	*F*(1,24) = 0.3	*F*(1,24) = 1.8
	*p* = 0.583	*p* = 0.195
Session	*F*(1,24) = 35.0	*F*(1,24) = 0.8
	*p* < 0.001^∗^	*p* = 0.387
Group × Round	*F*(2,24) = 2.4	*F*(2,24) = 3.4
	*p* = 0.116	*p* = 0.050^∗^
Group × Session	*F*(2,24) = 0.6	*F*(2,24) = 1.6
	*p* = 0.533	*p* = 0.228
Round × Session	*F*(1,24) = 0.4	*F*(1,24) = 0.3
	*p* = 0.537	*p* = 0.579
Three-way	*F*(2,24) = 0.5	*F*(2,24) = 0.2
	*p* = 0.586	*p* = 0.819


*Group refers to the between-subject factor with three levels (Anodal, Cathodal, or Sham). Session refers to a within-subject factor with two levels (pre- vs. post-production). Round refers to a within-subject factor with two levels (reflecting the 2 days during which participants came). ^∗^*p* < 0.05.*

This analysis revealed nothing new for thresholds: the effect of pre-/post-session (reflected by the leftward shift) did not interact with round or group. For slopes, there was a modest interaction between round and group, reflecting that round had an effect for Anodal [*F*(1,24) = 8.9, *p* = 0.006; slopes being 23.6% steeper in round 2 than in round 1] but not for Cathodal [*F*(1,24) < 0.1, *p* = 0.936] or Sham [*F*(1,24) = 0.2, *p* = 0.673], as illustrated by the most-right bottom panel of **Figure 5**.

From a qualitative perspective, the leftward shift seemed smaller in round 2 than in round 1 for the Anodal group. Thus, it would seem that Anodal tDCS could have enhanced the pull toward “head” during round 1 compared to the same subjects in the absence of tDCS in round 2. However, the lack of three-way interaction for thresholds in **Table 2** prevents us from drawing such a conclusion. Instead, those subjects exhibited steeper slopes in round 2, for both pre- and post-production sessions. Therefore, the potential effect of Anodal tDCS on the phonetic boundary between /e/ and /a/ in this study appears to be more complex than a horizontal shift of the entire psychometric function toward the vowel being repeated.

### Relation between Production and Perception

We now turn to the relation between the size of the adaptation effect in production with the leftward shift observed in perception. If anodal tDCS acted to exacerbate the pull toward “head” (as suggested by the within-subjects comparisons of round 1 and 2, in **Figure 5**), then we should find that the subjects showing the largest adaptation in F1 were the subjects showing the largest leftward shifts.

**FIGURE 6:**
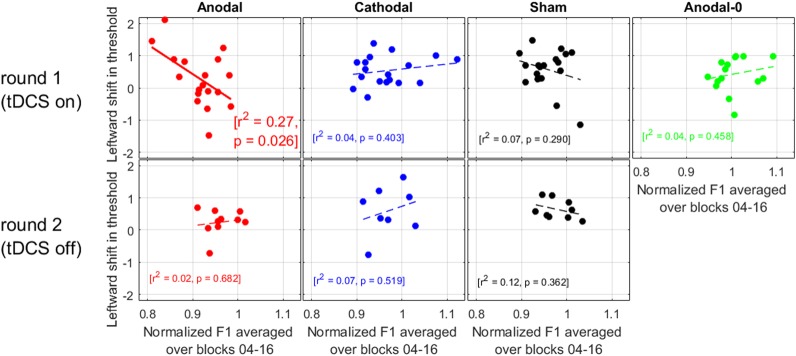
Relation between the leftward shift in threshold (perception data) and the normalized F1 values (production data) averaged over the ramp and hold phase.

As illustrated in **Figure 6**, there was no correlation for any of the groups during round 2, nor was there a correlation for the Cathodal, Sham, or Anodal-0 groups during round 1, but there was a significant correlation for the Anodal group in round 1. At the very least, this strengthens the argument that there is a genuine link between production data and perception data in this study, because the effects of interest occurred only for the subjects allocated to the Anodal group. More specifically, the subjects who exhibited the largest adaptation effect (i.e., the lowest F1 values produced in response to the F1 upward shift in auditory feedback) were the ones who had shifted their /e/-/a/ boundary the most leftward. In other words, this is consistent with the idea that anodal tDCS did act to enhance the pull toward the vowel /e/ being repeated.

### RT Data

**FIGURE 7:**
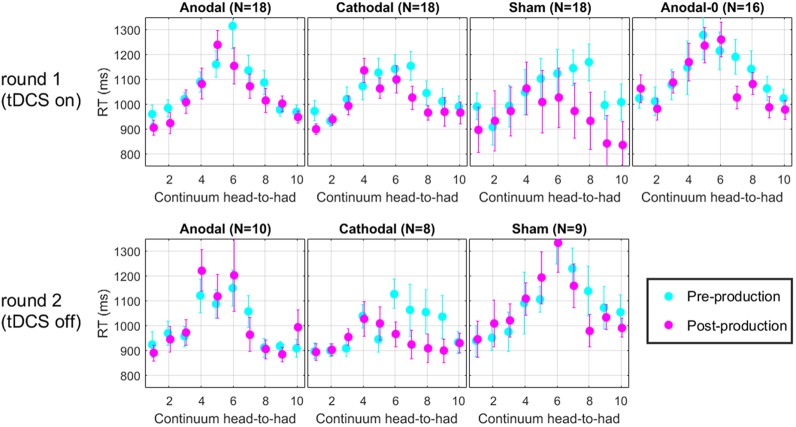
Mean reaction time along the perceptual continuum. Subjects took more time to respond when they faced ambiguous vowel percepts, which is indicative of a diligent behavior.

For each trial of the perceptual task, reaction time (RT) was recorded. **Figure 7** shows the RT along the continuum, for each group in the two rounds. Across all panels, it is clear that RT increases in the middle of the continuum. This effect arises simply because subjects took a bit more time to make their decision when they listened to very ambiguous vowel percepts such as those that corresponded to their 50% point on the psychometric function. It is tempting to think that one could get an idea of the perceptual boundary from the peak of these RT data. Indeed, the peak seems to shift leftward in the post-production RT compared with the pre-production RT. However, this measure does not appear sufficiently precise to lead to any new insights on the matters discussed above. We should only regard those data as a validation that subjects performed the perceptual task attentively since they took additional time when facing challenging stimuli, which is a classic pattern in the RT literature (Swensson, 1972; Luce, 1986).

## Discussion

### Mixed Success of tDCS

The present study provided solid evidence (both from between-groups comparisons and within-subject comparisons) that anodal tDCS of the IPL can successfully enhance the adaptation phenomenon in response to a F1 alteration. However, we failed to find any evidence that cathodal tDCS could do the contrary, i.e., hinder the adaptation to the feedback alteration in F1, since the effects observed in this configuration never differed from sham stimulation or even when the tDCS equipment was absent. Therefore, we must conservatively conclude that our overall hypothesis that tDCS can be utilized to modulate speech motor learning received mixed success in this study: it can only go in one direction.

This directionality, however, is not uncommon in the emerging literature on tDCS. Most convincingly, Jacobson et al. (2012) conducted a meta-analytical review of those polarity effects for motor and cognitive functions. Studies (15 of which used both anodal and cathodal configurations) looking at motor functions have generally found symmetrical behaviors: anodal tDCS being associated with excitatory effects and cathodal tDCS being associated with inhibitory effects. In contrast, studies (19 of which used both configurations) looking at cognitive functions have generally found asymmetrical behaviors: anodal tDCS being associated with facilitation of performance of the stimulated area but cathodal tDCS being largely ineffective. In fact, studies related to language were particularly immune to inhibitory cathodal effects. Therefore, the present results are very much in line with the asymmetrical pattern reviewed by Jacobson et al. (2012) for the effect of tDCS polarity on cognitive functions. One interpretation for this asymmetry is that cognitive functions, and particularly those involved in speech and language, are often supported by large and intricate networks (Catani et al., 2005). Some of them could be unaffected by the inhibition induced by cathodal tDCS. Alternatively, cathodal stimulation effects in motor studies have been applied over motor areas. The stimulation area in the present study (left IPL) is not an area involved in motor control but rather sensorimotor integration. However, Shum et al. (2011) did successfully inhibit speech motor learning using rTMS. One important difference to bear in mind is that there is significant current spread for tDCS compared to the more focal effects associated with rTMS (Williams et al., 2009; Buch et al., 2016). In addition, tDCS is only neuromodulatory while TMS both stimulates and modulates and thus will force action potentials (which tDCS will not). Finally, in contrast to off-line rTMS where the adaptation paradigm is conducted following the stimulation, in current approaches using tDCS, the stimulation occurs during the task. Task-related stimulation may be distributed into additional network components which may engage multiple brain areas that are not affected for off-line stimulation. Overall, it is not surprising that tDCS and rTMS may result in different effects. In the context of the current study in which the altered feedback resulted in an increase in the contribution (engagement) of the IPL, it appears the inhibitory neuromodulation was not strong enough to offset the increased excitation engaged as a result of the adaptation.

### Speech Motor Learning and Speech Motor Control

A secondary hypothesis we addressed was whether neuromodulatory effects on the IPL would be observed in the absence of an error—that is when there was no accompanying feedback manipulation. The lack of change in speech production under excitatory stimulation in the presence of normal auditory feedback suggests that the IPL may not be engaged for normal speech production, but only engaged for new, novel or adaptive conditions. Consistent with this interpretation, regions of the left IPL that were targeted here and in Shum et al. (2011), often show deactivation in functional neuroimaging studies of speech production (Dhanjal et al., 2008; Geranmayeh et al., 2012, 2014). Interestingly, this brain region is a part of the default mode network which is characteristically deactivated for speech production (Dhanjal et al., 2008; Seghier and Price, 2012). In the present study, the lack of effect of anodal stimulation under normal feedback, similar to the lack of effect under rTMS in the absence of an induced speech error (Shum et al., 2011) is consistent with the IPL being inactive (or even deactive) under normal speech production in which well-learned patterns are produced. This interpretation is also consistent with the function of the posterior parietal cortex as an integral component for the formation of internal models of motor control (Wolpert and Kawato, 1998; Desmurget and Grafton, 2000; Della-Maggiore et al., 2004). The IPL receives multisensory input from the dorsal auditory stream as well as projections from somatosensory cortex (predominantly feedback), feed-forward projections from premotor and inferior frontal cortices (Rauschecker and Scott, 2009; Rauschecker, 2010) and targeted output from the cerebellum (Clower et al., 2001) to facilitate sensorimotor prediction and induce sensorimotor plasticity (Blakemore and Sirigu, 2003). Overall the IPL is an important network component for imitation and subsequent learning (Rizzolatti et al., 2006; Molenberghs et al., 2009). For speech production, it appears that when well-learned speech motor patterns do not suffice, an additional network is recruited to update the sensorimotor internal model suggesting that the neural substrate and mechanisms for speech motor control and speech motor learning, while overlapping, are not mutually inclusive.

Clinical populations often exhibit abnormal sensorimotor integration and reduced capacity for re-learning. For example, Mollaei et al. (2013) used a paradigm very similar to that presented here and showed that patients with Parkinson’s disease do not respond to altered feedback as much as a control group of speakers. The reduced ability to adapt their speech is consistent with their well-known resistance to clinical treatment focused on remediating their speech disorder. For such patients, it might be useful to use anodal tDCS to attempt to normalize sensorimotor adaptation to enhance their speech rehabilitation.

### Malleability of Perceptual Representation

It is known that repeated exposure to a stimulus within a perceptual continuum can result in a narrowing of this stimulus category, also referred to as the “selective adaptation effect” (Eimas and Corbit, 1973). In our study, this translated into fewer stimuli being recognized as “head,” i.e., a shift in the phonetic boundary toward “head.” We therefore expected subjects in the Anodal-0 group (receiving unaltered feedback) to exhibit this behavior and this is exactly what we found. For the subjects receiving altered feedback, we had less expectation (let alone any compounding effect of tDCS). For example, Shiller et al. (2009) used a continuum between /s/ (as in “see”) and /sh/ (as in “she”). Following repetitive production of the /s/ sound, subjects with unaltered feedback shifted their boundary toward /s/, consistent with the classic selective adaptation effect. However, following repetitive production of the /s/ sound with feedback altered toward /sh/, subjects shifted their boundary toward /sh/. In our study, this would have translated into a shift in the direction of the F1 shift (+30%, toward “had”). We did not find this pattern in any group, suggesting that there may be something inherently different in the perceptual malleability of vowels with that of sibilant fricatives in an adaptation paradigm.

Another, particularly relevant, study was performed by Lametti et al. (2014) using the same continuum “head-to-had” as ours. They used two groups of subjects: the first one (similar to here) was adapted to an upward-shift in F1 and consequently lowered their F1 productions by about 50 Hz (∼8%); and the second one was adapted to a downward-shift in F1 and consequently increased their F1 productions by about 50 Hz (∼8%). They measured perceptual changes at 50% for pre- and post-adaptation sessions and found that perceptual shifts only occurred for the second group (by about 0.35), not the first. This latter result is inconsistent with our study, in which subjects were adapted to an upward-shift in F1 and exhibited a significant perceptual shift (ranging from 0.2 to 0.6). In a second experiment, Lametti et al. replicated their design but measured the perceptual thresholds within the “hid-to-head” continuum (“hid” having an even lower F1 than “head”). They found that perceptual shifts occurred for the first group (by 0.75), and to a smaller degree for the second (by about 0.25). They concluded that, in both experiments, the changes in perceptual space were greater when the continuum used for testing was within the range of F1 values produced, not the range of F1 values perceived (during the adaptation task). In other words, the selective adaptation effect would pull more on the side of speech production than on the side of auditory feedback. Hence, their result provided support for the motor theory view of speech perception (Liberman et al., 1967). In the light of their results, it seems certainly plausible that we could have observed larger perceptual shifts (still toward “head,” the stimulus being repeated) had we measured those thresholds within the “hid-head” continuum rather the “head-had” continuum. But one should certainly not conclude that the phonetic range covering the auditory feedback is rigid: 70 subjects in round 1 and 27 subjects in round 2 exhibited a typical shift in the present study. Even though the effect size is relatively small (a shift of 0.4 on average over the 01–10 continuum), it was here very consistent among groups, with N as little as 8, so it is presumably not just a matter of statistical power. There are massive individual differences not only in the size of the adaptation phenomenon (Lametti et al., 2012), but also on the size of the perceptual shifts that result from it. Caution should be exerted when comparing one study to another, simply on the basis of this inter-individual variability. One result, for example, that is surprising in Lametti et al.’s study is the absence of perceptual shift for subjects who received unaltered feedback (eventually similar to our Anodal-0 group since the tDCS did not appear to do anything in this case). One could have expected the mere repetition of the word “head” to shrink its category, in line with the classic selective adaptation effect, but it did not happen in their study.

We also did not find a correlation between the amount of compensation and the shift in phonetic boundary for six out of seven cases (**Figure 6**). This lack of correlation had also been found by Shiller et al. (2009), Lametti et al. (2014), and in other modalities (e.g., visuo-motor adaptation, Cressman and Henriques, 2009), but it has been established with alterations in somatosensory feedback in the context of speech motor learning (Nasir and Ostry, 2009). So, this relationship is another matter of debate. In the present study, this correlation was established only for the subjects who received anodal tDCS: those exhibiting the largest adaptation to the F1 alteration were those exhibiting the largest shift toward “head.” This result seems more consistent with the idea proposed by Lametti et al. (2014): indeed, all three groups (Anodal, Cathodal, and Sham) received feedback that was altered to the same degree; but the F1 productions of subjects in the Anodal group were particularly lowered compared to the other two groups. If it is production that drives perceptual recalibration, one could then have expected the Anodal subjects to exhibit the largest perceptual changes. This was not the case when comparing between groups (**Figure 4**), but it trended in the expected direction when comparing Anodal subjects between the two rounds (**Figure 5**), and it was the case when comparing individuals within the Anodal group (**Figure 6**).

## Summary

The present study investigated the potential use of tDCS as a tool to modulate speech motor learning in healthy adults. Under conditions of altered auditory feedback, subjects exhibited an increase in adaptation to anodal-tDCS of the IPL but exhibited no decrease in adaptation using cathodal-tDCS compared to the sham condition. Under conditions of anodal stimulation in the absence of altered feedback, no change in production was observed. In addition, the phonetic boundary between /e/ and /a/ shifted toward the vowel /e/ being repeated in all groups of subjects and this perceptual shift was slightly enhanced by the anodal-tDCS. The motor effects of modulating the left IPL is dependent on whether the production task requires speech motor learning but is minimally engaged for speech motor control. Knowing the contribution (activation, no activation, or deactivation) of a targeted brain area to a specific task is critical in deciding whether and what kind of neuromodulation to apply.

## Author Contributions

MD collected 36% data, analyzed the data, and wrote the manuscript. DN collected 64% of the data and contributed to data analysis. VG led the development of the rationale and conceptualization of the project, designed the experiments and contributed to manuscript writing.

## Conflict of Interest Statement

The authors declare that the research was conducted in the absence of any commercial or financial relationships that could be construed as a potential conflict of interest.
